# POPs and Gut Microbiota: Dietary Exposure Alters Ratio of Bacterial Species

**DOI:** 10.1289/ehp.123-A187

**Published:** 2015-07-01

**Authors:** Carol Potera

**Affiliations:** Carol Potera, based in Montana, also writes for *Microbe*, *Genetic Engineering News*, and the *American Journal of Nursing*.

Persistent organic pollutants (POPs) have been implicated in myriad human health problems, including cancer, neurologic, immunologic, and reproductive defects, among many other adverse health effects.^1^ New lines of research suggest that chronic dietary exposure to POPs may also contribute to obesity and type 2 diabetes.^2^ In this issue of *EHP*, researchers examine how one POP in particular—2,3,7,8 tetrachlorodibenzofuran (TCDF)—affects the composition of the mouse gut microbiome.^3^ They report that TCDF exposure alters the gut microbiome in ways that may prove to contribute to obesity and other metabolic diseases.

TCDF binds the aryl hydrocarbon receptor (AHR), which activates a variety of biological responses.^1^ Recent studies indicate that keeping the gut in good working order is one of these functions.^4^

**Figure:**
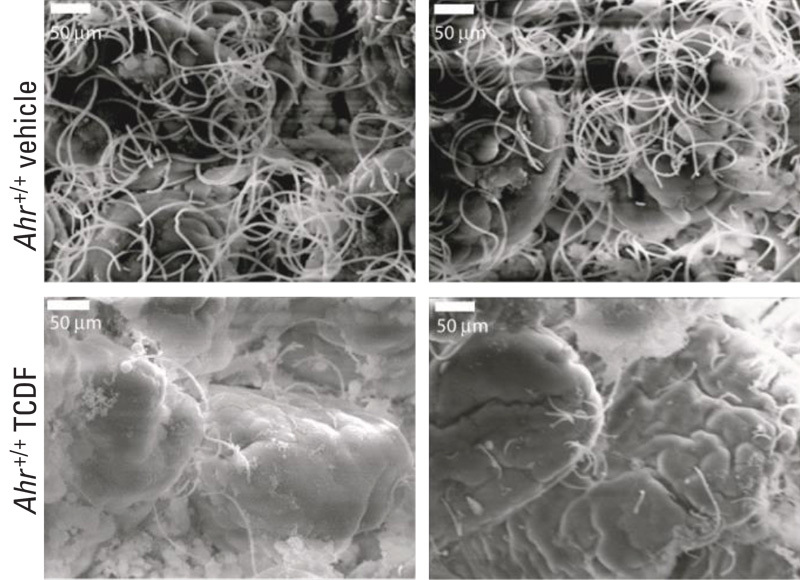
Scanning electron microscope images show dense networks of bacteria in the small intestines of control mice (top), which were dramatically depleted in the small intestines of TCDF-treated mice (bottom). Source: Zhang et al. (2015)^3^

The gut microbiome is increasingly being recognized as influential in multiple aspects of health. “We’ve been focused on how POPs impact the host. Now we’re starting to see how these chemicals may impact the gut bacteria, either positively or negatively,” says study leader Andrew Patterson, an assistant professor of molecular toxicology at Pennsylvania State University.

For five days, Patterson’s team fed food pellets spiked with 24 µg/kg TCDF to adult male mice that either carried or lacked the *AhR* gene. This helped to confirm that the AHR truly is involved in regulating the end points measured, including fluctuations in gut microbe populations, liver enzymes, and bile acids.

Mice that lacked the *AhR* gene underwent marked changes in their gut microbial populations, with a decrease in Firmicutes species and an increase in Bacteroides species. These changes were associated with significantly increased levels of bile acids and short-chain fatty acids, altered liver function, increased intestinal inflammation, and inhibited signaling of the farnesoid X receptor, a key regulator of fat and glucose metabolism.^3^

The mice received a very high dose of TCDF, equivalent to 3,000 ng/kg in people.^3^ That’s about 1,000 times more than most adults are thought to ingest each day through food^5^ but in the range of high-dose industrial exposures.^6^ The researchers chose this high dose to ensure they would find observable changes and end points to pursue further. Now they’re feeding mice lower chronic doses of TCDF, and according to Patterson, preliminary data are showing similar changes as those seen at higher TCDF doses.

Patterson’s results “show significant changes in the gut microbiome profile after TCDF administration, and they raise very interesting and important questions. But exactly what that means downstream needs more work to answer,” says Stephen Safe, a distinguished professor of toxicology at Texas A&M University, who was not involved with the study. The results add to mounting evidence that changes in the gut microbiome modulate many different diseases, Safe says.

The results also contribute to the growing evidence that the gut microbiome is an important, although still not fully understood, player in the toxicity of environmental pollutants, says Michal Toborek, a professor and vice chair for research at the University of Miami Miller School of Medicine. Toborek previously reported in *EHP* that high doses of polychlorinated biphenyls, another type of POP, altered the composition of the gut microbiome in mice but that exercise appeared to blunt this effect.^7^
